# Supplementary material for: HIVprotI: an integrated web based platform for prediction and design of HIV proteins inhibitors
Source: J Cheminform. 2018 Mar 9;10:12. doi: 10.1186/s13321-018-0266-y (PMC5845081; doi:10.1186/s13321-018-0266-y)
Supplement: Supplementary file 2 — Additional file 2. Source code of HIVProtI web server. [file 13321_2018_266_MOESM2_ESM.zip › HIVprotI_Source-code/hivprotI/weka-3-6-6/doc/weka/classifiers/evaluation/Prediction.html]

Prediction


---


|  |  |  |
| --- | --- | --- |
| **Overview** \| **Package** \| **Class** \| **Tree** \| **Deprecated** \| **Index** \| **Help** \| **Weka's home** | |  |
| **PREV CLASS**   **NEXT CLASS** | **FRAMES**    **NO FRAMES**     **All Classes** |  |
| SUMMARY: NESTED \| FIELD \| CONSTR \| METHOD | DETAIL: FIELD \| CONSTR \| METHOD |  |


---


## weka.classifiers.evaluation Interface Prediction

**All Known Implementing Classes:**: NominalPrediction, NumericPrediction

---

``` public interface Prediction ```

Encapsulates a single evaluatable prediction: the predicted value plus the
actual class value.

**Version:**
:   $Revision: 1.7 $

**Author:**
:   Len Trigg (len@reeltwo.com)

---

| **Field Summary** | |
| --- | --- |
| `static double` | `MISSING_VALUE`             Constant representing a missing value. |


| **Method Summary** | |
| --- | --- |
| `double` | `actual()`             Gets the actual class value. |
| `double` | `predicted()`             Gets the predicted class value. |
| `double` | `weight()`             Gets the weight assigned to this prediction. |

**Field Detail**

### MISSING\_VALUE

```
static final double MISSING_VALUE
```

:   Constant representing a missing value. This should have the same value
    as weka.core.Instance.MISSING\_VALUE


**Method Detail**

### weight

```
double weight()
```

:   Gets the weight assigned to this prediction. This is typically the weight
    of the test instance the prediction was made for.

    :   **Returns:**: the weight assigned to this prediction.

---


### actual

```
double actual()
```

:   Gets the actual class value.

    :   **Returns:**: the actual class value, or MISSING\_VALUE if no prediction was made.

---


### predicted

```
double predicted()
```

:   Gets the predicted class value.

    :   **Returns:**: the predicted class value, or MISSING\_VALUE if no prediction was made.


---


|  |  |  |
| --- | --- | --- |
| **Overview** \| **Package** \| **Class** \| **Tree** \| **Deprecated** \| **Index** \| **Help** \| **Weka's home** | |  |
| **PREV CLASS**   **NEXT CLASS** | **FRAMES**    **NO FRAMES**     **All Classes** |  |
| SUMMARY: NESTED \| FIELD \| CONSTR \| METHOD | DETAIL: FIELD \| CONSTR \| METHOD |  |


---
